# Corrigendum to “Safety and Clinical Usage of Newcastle Disease Virus in Cancer Therapy”

**DOI:** 10.1155/2017/4529437

**Published:** 2017-12-03

**Authors:** Han Yuen Lam, Swee Keong Yeap, Mehdi R. Pirozyan, Abdul Rahman Omar, Khatijah Yusoff, Suraini Abd-Aziz, Noorjahan Banu Alitheen

**Affiliations:** ^1^Department of Cell and Molecular Biology, Faculty of Biotechnology and Biomolecular Sciences, Universiti Putra Malaysia, 43400 Serdang, Selangor, Malaysia; ^2^Institute of Bioscience, Universiti Putra Malaysia, 43400 Serdang, Selangor, Malaysia; ^3^Department of Microbiology, Faculty of Biotechnology and Biomolecular Sciences, Universiti Putra Malaysia, 43400 Serdang, Selangor, Malaysia; ^4^Department of Bioprocess Technology, Faculty of Biotechnology and Biomolecular Sciences, Universiti Putra Malaysia, 43400 Serdang, Selangor, Malaysia

In the article titled “Safety and Clinical Usage of Newcastle Disease Virus in Cancer Therapy” [1] the name of the third author was given incorrectly as Mehdi Rasoli. The author's name should have been written as Mehdi R. Pirozyan. In addition, the first and last names of the sixth author were reversed. The revised authors' list is shown above.
